# Management of polyneuropathy using yoga and naturopathic medicine in India: recommendations for future research and clinical practice

**DOI:** 10.3389/fpain.2023.1264450

**Published:** 2023-10-25

**Authors:** Pradeep M. K. Nair, Karishma Silwal, Jyoti Keswani, Sucheta Kriplani, Vakeel Khan, Ayush Maheshwari, Mili Arpan Shah, Naga Jyoti, Vinutha Rao, Cijith Sreedhar, Kinjal Dilipsinh Bhalavat, Renjish Mohanan, Jerin Subha M, Rakesh Gupta, Hemanshu Sharma, Gulab Rai Tewani

**Affiliations:** ^1^ Department of Integrative Oncology and Research, Mirakle Integrated Health Centre, Pollachi, India; ^2^ Department of Naturopathy, Sant Hirdaram Yoga and Nature Cure Hospital, Bhopal, India; ^3^ Department of Yoga, Sant Hirdaram Medical College of Naturopathy and Yogic Sciences for Women, Bhopal, India; ^4^ Department of Yoga, Sant Hirdaram Yoga and Nature Cure Hospital, Bhopal, India; ^5^ Department of Anatomy, Sant Hirdaram Medical College of Naturopathy and Yogic Sciences for Women, Bhopal, India; ^6^ Department of Holistic Medicine, Body Holiday Resort, Cap Estate le Sport, Saint Lucia; ^7^ Department of Ozone Therapy, Ozone Forum of India, Mumbai, India; ^8^ Department of Yoga, Naturopathy and Acupuncture, Dr. Jyoti's Nature Cure Clinic, Bangalore, India; ^9^ Department of Yoga and Naturopathy, MVM College of Naturopathy and Yogic Sciences, Bangalore, India; ^10^ Department of Yoga and Naturopathy, Prakriti Shakti Clinic of Natural Medicine, Iduki, India; ^11^ Department of Physiology, Morarji Desai Institute of Naturopathy and Yoga, Vadodara, India; ^12^ Department of Massage, Nandha Naturopathy and Yoga Medical College, Erode, India; ^13^ Department of Physiology, Nandha Naturopathy and Yoga Medical College, Erode, India; ^14^ Department of Yoga and Naturopathy, University College of Naturopathy and Yogic Sciences, Jodhpur, India; ^15^ Department of Community Medicine, Sant Hirdaram Medical College of Naturopathy and Yogic Sciences for Women, Bhopal, India; ^16^ Department of Yoga and Naturopathy, Sant Hirdaram Yoga and Nature Cure Hospital, Bhopal, India

**Keywords:** polyneuropathy, yoga, naturopathy, complementary medicine, peripheral neuropathy

## Introduction

Yoga and naturopathy is one of the official indigenous systems of medicine in India under the Ministry of Ayurveda, Yoga and Naturopathy, Unani, Siddha, Sowa-Rigpa, and Homoeopathy (AYUSH), Government of India (1). Yoga and naturopathy is widely used as a lifestyle medicine for almost all systemic disorders, like musculoskeletal disorders, metabolic disorders, autoimmune disorders, neurological disorders, skin disorders, cardiovascular disorders, and respiratory disorders (2–4). Yoga and naturopathy use a holistic, patient-centered approach to treating various conditions. Hydrotherapy, therapeutic fasting, diet therapy, yoga therapy, mud therapy, heliotherapy, chromotherapy, magnetotherapy, physiotherapy, ozone therapy, acupressure, and acupuncture are the commonly used therapeutic modalities in yoga and naturopathic medicine (5). These therapies are used in combination with varying frequency as per the needs or underlying conditions of the patients. As discussed earlier, yoga and naturopathy physicians treat an array of clinical conditions, including polyneuropathies (PN), a generalized disorder of the peripheral nervous system. In our practice, we encounter PN as a comorbid condition, usually presenting along with another systemic disorder, most commonly type 2 diabetes mellitus.

## Yoga and naturopathy approach in PN

India is one of the few countries in the world that operates stand-alone, state-regulated inpatient hospitals and medical colleges offering yoga and naturopathy interventions. In India, yoga and naturopathy is practiced as a holistic medical system following the international guidelines of naturopathic medicine such as: The Healing Power of Nature (Vis *Medicatrix Naturae*), Identify and Treat the Causes (*Tolle Causam*), First Do No Harm (*Primum Non Nocere*), Doctor As Teacher (*Docere*), Treat the Whole Person (*Tolle Totum*), and Prevention (Preventare) (6). The clinical presentation of PP can be classified into four major categories: (1) sensory symptoms (pain, loss of sensation, burning sensation, and ulcers); (2) motor symptoms (gait instability, muscle cramps, and paresis); (3) autonomic symptoms (digestive disturbances, urogenital symptoms, bladder dysfunction, tachycardia, blood pressure changes, and dryness in the skin); and (4) psychological symptoms (stress, quality of life disturbances, and sleep disturbances).

Yoga and naturopathy physicians follow a three-pronged approach in the management of all diseases, including PN, where they classify their treatments into eliminative therapies, conservative therapies, and sustainable therapies. Eliminative therapies stand for those treatments that are meant to promote elimination in the major eliminative organs like the lungs, intestine, skin, and kidney, as well as the mind, aimed at addressing the root cause, whereas conservative therapies attempt to offer symptomatic care, and lastly, sustainable therapies intend to build salutogenic health resources by offering permanent lifestyle measures (6–9). Supplementary Table S1 provides an exhaustive list of the therapies employed by yoga and naturopathy physicians in Indian settings to treat PN.

## Scientific basis of yoga and naturopathic interventions for PN

Even though the evidence base literature suggesting the scientific rationale and clinical utility of yoga and naturopathic modalities is increasing over the past years, studies drawing direct inferences on the usefulness of yoga and naturopathy interventions are scarce. Hydrotherapy integrated with massage has been shown to improve the nerve growth factor, postural stability, and blood sugar levels in patients with diabetic neuropathy (10). Contrast baths have been shown to reduce neuropathic pain among patients with diabetic neuropathy (11, 12). Secondary literature suggests that hydrotherapy modalities like cryotherapy, packs, steam bath, sauna bath, head-out immersion, sitz bath, and enema to be useful in the management of various clinical conditions including PN (13, 14).

Mud therapy is another popular prescription in the management of PN. Thermal mud baths have been shown to improve the quality of life and clinical symptoms associated with diabetic polyneuropathy (15). A recent case report reported the beneficial effect of the combined yoga and naturopathy protocol in alleviating pain and improving quality of life among PN patients (16). Mooventhan et al., in their comprehensive review investigating the neuroprotective effect of yoga, have highlighted several beneficial effects of yoga in alleviating pain and improving the nerve conduction velocity in PN (17–20). Jinny et al. demonstrated that yoga therapy improves postural stability and balance in patients with PN (21). Besides this, other mind-body interventions like Reiki and meditation practices have also been shown to reduce neurotoxicity and improve the quality of life among patients with chemotherapy-induced peripheral neuropathy (22).

Fasting therapy, another commonly used modality in yoga and naturopathic practice, is also demonstrated to improve nerve function and associated symptoms by attenuating the oxidative stress and underlying metabolic disarrays in PN (23–25). Diet therapy plays a major role in yoga and naturopathic medicine-based regimens for all diseases, including PN. Plant-based diets have been shown to improve glycemic control, reduce tissue hypoxia, improve endoneurial microvasculature perfusion, and alleviate pain in patients with PN (26). Recent studies investigating the role of acupuncture in improving the clinical outcomes of PN have shown acupuncture to improve sensory and motor nerve conduction and also reduce neuropathic symptoms. Acupuncture modulates nerve growth factor signaling, attenuates inflammation, and other factors like G-protein-coupled receptor 78, purinoceptors, etc. that are associated with symptoms of PN (27, 28). Similar reports are available demonstrating the possible use of acupressure in the management of PN, especially chemotherapy-induced neuropathy and diabetic neuropathy (29, 30).

Indirect evidence indicates sun exposure (heliotherapy) to be a potential tool in modulating the cardiometabolic dysfunction that is postulated to be a risk factor for PN (31). However, there is no direct evidence available to suggest the usefulness of sun exposure, even though there are studies linking the lack of vitamin D in PN (32), for which sun exposure is a primary source. Physiotherapy treatments like exercise therapy and electrotherapy are used as an integral component of yoga and naturopathy protocols for PN. This is commonly indicated to treat the pain, muscle weakness, balance impairment and postural dysfunctions (33). Evidence suggests physiotherapy reduces the symptoms and improves the quality of life among patients with chemotherapy-induced peripheral neuropathy and diabetic polyneuropathy (33–35).

**Figure 1:**
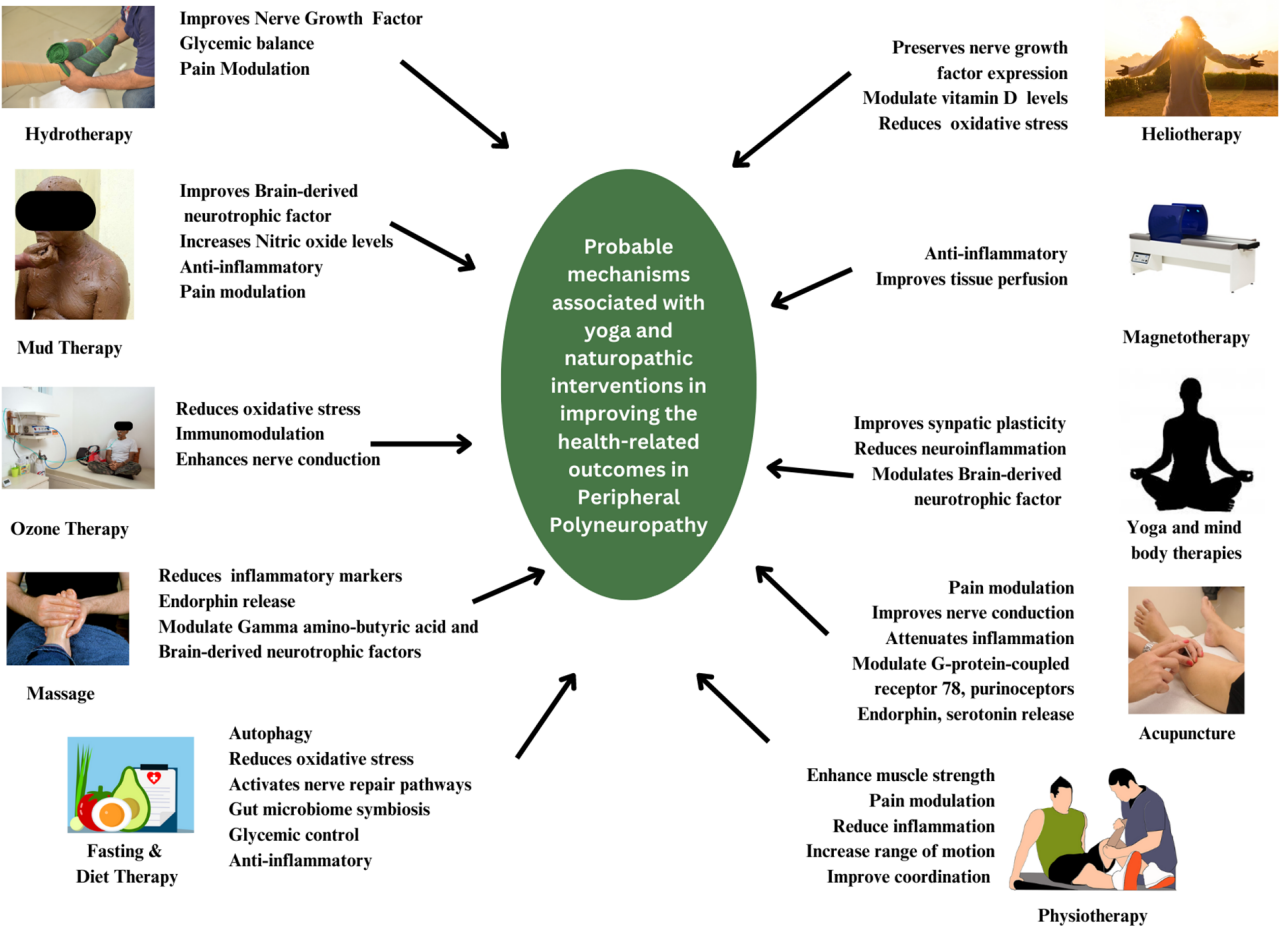
Yoga and naturopathy therapies' potential biological pathways of action in polyneuropathy.

Ozone therapy, the use of ozone molecules generated from medical-grade oxygen in varying doses and forms is a well-known anti-oxidant, antibiotic, and analgesics agent (36). Numerous studies suggests the usefulness of ozone therapy in modulating the pain sensation, improving nerve conduction and reduce the symptoms associated PN (37, 38). Apart from this, a recent systemic scoping review of the treatment options for Chemotherapy-Induced Peripheral neuropathy identified numerous supportive therapies like aromatherapy, hydrotherapy, diet therapy, massage, acupuncture, acupressure, yoga therapy, and herbal medicine to have moderate to high clinical efficacy (39). Figure 1 summarizes the possible mechanisms by which yoga and naturopathic medicine therapies may attenuate the symptoms associated with PN.

## Future directions for clinical practice and research

Yoga and naturopathic medicine in India is an evidence-based practice that include a conglomerate of therapies that are drugless and holistic in nature (5). However, direct evidence demonstrating the usefulness of yoga and naturopathic medicine, as practiced in India, in the management of PN is not available in the published literature. While the user base of yoga and naturopathy is growing in India, the lack of scientific literature in PN from Indian settings depicts a serious issue of underreporting, a lack of knowledge in research and publication procedures, and a lack of motivation from public enterprises to promote research in yoga and naturopathy. Besides, some therapies used in yoga and naturopathy for PN, like chromotherapy and magnetotherapy, are solely based on anecdotal experiences or non-peer-reviewed textbook knowledge. While these therapies have shown good clinical potential, a lack of documentation harms their scientific appeal and their effective use among all stakeholders.

As discussed, yoga and naturopathy physicians in India utilize a broad range of treatment choices to manage PN. However, the choice and utility of these therapies are primarily dependent on the individual physician's expertise, which is based on their personal experience and knowledge. There is a need to improve reporting practices among yoga and naturopathy physicians by offering high-quality training programs that would enable them to engage in evidence-based clinical practice. Given the disparities in practice patterns among Indian yoga and naturopathy physicians in the management of PN, expert committees or consensus groups that can pragmatically document clinical practices and recommend consensus guidelines to strengthen clinical practice and research are needed. Nevertheless, the present evidence hints at yoga and naturopathic medicine approaches as promising tool in the management of PN.
