# Supplementary material for: Management of polyneuropathy using yoga and naturopathic medicine in India: recommendations for future research and clinical practice
Source: Front Pain Res (Lausanne). 2023 Oct 25;4:1264450. doi: 10.3389/fpain.2023.1264450 (PMC10634222; doi:10.3389/fpain.2023.1264450)
Supplement: Supplementary file 1 [file Table1.docx]

| **Therapies**  **Recommended** | **Sensory symptoms**  ***Pain, burning***  ***loss of sensation*** | **Motor symptoms**  ***Gait instability***  ***Muscle cramps***  ***Paresis*** | **Autonomic symptoms**  ***Digestive disturbances***  ***Urogenital symptoms***  ***Bladder dysfunction***  ***Tachycardia and blood pressure*** | **Psychological symptoms**  ***Stress and poor quality of life***  ***Sleep disturbances*** |
| --- | --- | --- | --- | --- |
| **Hydrotherapy** |  |  |  |  |
| Packs | Arm pack  Leg pack | Arm pack  Leg pack | Abdomen packs  Kidney packs  Chest pack | Head pack |
| Baths | Arm bath  Leg baths  Contrast baths  Immersion baths  Sauna / steam bath | Arm baths  Leg baths  Sauna/ steam bath | Hip bath  Spinal bath | Immersion baths  Foot and arm baths  Cold Spinal baths |
| Compress | Warm compress to painful area | Warm compress to painful area | Cold compress to spine | Cold compress to eyes |
| Water irrigation | Enema  Douches | Enema  Douches | Enema  Spinal spray | Enema  Spinal spray |
| **Mud therapy** | Mud application to arms  Mud application to legs  Full body mud bath  Mud poultices in painful area | Mud baths  Mud application | Mud packs to abdomen  Mud application to chest  Mud application to spine | Mud pack to eyes  Full body mud bath |
| **Massage therapy** | Arm massage  Leg massage | Arm massage  Leg massage | Back massage  Abdominal massage | Head massage  Aroma massage |
| **Aroma therapy** | Cinnamon oil  Ginger oil  Tea tree oil  Rosemary oil | Sandal wood oil  Cypress oil | Peppermint oil  Bergamot oil | Lavender oil  Chamomile oil  Eucalyptus oil |
| **Acupuncture** | LI-4, LV-3, BL-10, GB-14, GB-20, ST-7, ST-8, ST-44. Weekly one fire cupping session with a duration of 15mins for 4 weeks has been seen beneficial. | Upper limbs- LU-5, PC-6.  Lower limbs- SP-6, SP-9,  SP-10, LV -8,  Scalp acupuncture. | BL-31, BL-32, BL-33, BL-28, BL -23, SP-6, ST-36, KD-3, H-7, PC-6. | Buddha’s triangle -  H-7, P-6, L-9. YinTang, SP – 5,  ST – 36. |
| **Acupressure** | ST-42, KD -3, LV-4, LI -4,  H-7, | BL-57, GV-26, LV-3, CV-4, KD-1, LU-7, LU-9 | BL-10, EX-3, RN -17, PC-6,  HT -7, LV-3, SP-6, ST-36,  CV-12, BL-21, CV-6. | DU -20, Gb-21, H-7, SP-6, LV-3, K-1,  SI -3. |
| **Herbs** | Gingko Biloba  Ocimum sanctum  Ashwagandha(Withania somnifera)  Vacha (Acorus calamus) | Gokhru  Occimum sanctum  Ginger  Ashwagandha(Withania somnifera | Saffron  Cinnamon  Ashwagandha(Withania somnifera)  Triphala | Shatavari (Asparagus racemosu)  Brahmi (Bacopa monnieri)  Chamomile |
| **Fasting** | Intermittent fasting  Short / prolonged fasting | Intermittent fasting  Short / prolonged fasting | Intermittent fasting  Short / prolonged fasting | Intermittent fasting  Short / prolonged fasting |
| **Diet Therapy** | Vegetarian diet consisting of whole grains, nuts, seeds, sprouts,seasonal fruits and vegetables | Vegetarian diet consisting of whole grains, nuts, seeds, sprouts, seasonal fruits and vegetables | Vegetarian diet consisting of whole grains, nuts, seeds, sprouts, seasonal fruits and vegetables | Vegetarian diet consisting of whole grains, nuts, seeds, sprouts, seasonal fruits and vegetables |
| **Yoga Therapy** |  |  |  |  |
| *Yoga asanas* | Tadasana (Mountainpose),  Bhujangasana (Cobra pose)  Anadabalasana (Happy baby pose)  Shalabhasana (Locust pose)  Dhanurasana (Bow pose)  Trikonasana (Triangle pose)  Pawanmuktasana (Wind relieving pose) | Tadasana (Mountain pose)  Shavasana (Corpse pose)  Supported SuptaBaddhaKonasana (Cobbler’s pose)  Setubandhasana (Bridge pose) Markatasana (Supine Spinal twist pose) | Katichakrasana (Standing spinal twist)  Shalabhasana (Locust pose)  Dhanurasana (Bow pose)  Pawamuktasana (Wind relieving pose)  Ardhchakrasana (Half wheel pose) | Shavasana (Corpse pose)  Padmasana (Lotus pose) |
| *Loosening exercises* | Loosening exercises | Loosening exercises | Loosening exercises | Loosening exercises |
| *Pranayama* | Deep breathing  AnulomViloma Pranayama  Bhramari | AnulomViloma Pranayama, Bhramari  Bhastrika | AnulomViloma Pranayama  Bhramari | AnulomViloma Pranayama |
| *Dhyana and relaxation techniques* | Yoga Nidra  Pranic Energization Technique  AUM mantra chanting | Mantra Meditation  Mindfulness meditation | Sound healing  Yoga Nidra | Yoga Nidra  AUM Meditation  Love and kindness meditation |
| **Helio therapy** | Sun exposure to hands and feet  Banana leaf bath | Sun exposure of hands, feet and back | Sun exposure of whole spine | Sun exposure of hands, feet and spine |
| **Chromo therapy** | Red and Orange colour in the form of infrared radiation, charged oil and water) | Red, Orange and Yellow colour in the form of infrared radiation, charged oil and water) | Yellow, Green and orange colour in the form of charged water, fruits and vegetables. | Blue light exposure  Head massage with blue charged oil.  Blue color visualization. |
| **Magneto therapy** | South pole magnets can be directly applied or given in the form of charged water.  Magnetic belt to painful areas. | Lead-V application; North pole to right foot and South pole to left foot. | Lead -II application; North pole to right hand and South pole to left foot. North pole charged water for drinking. | Lead I application; North pole to right hand and south pole to the left hand |
| **Ozone Therapy** | Rectal insufflation  Breathing Ozone through Oil  Bagging / funneling in affected areas  Minor autohemotherapy  Intramuscular injections  Ozone steam and sauna | Rectal insufflation  Breathing Ozone through Oil  Bagging / funneling in affected areas  Minor autohemotherapy  Intramuscular injections  Subcutaneous injections | Rectal insufflation | Ear insufflation  Breathing Ozone through Oil |
| **Physiotherapy** | Muscle stimulation  Contrast fomentation  TENS  Moist Heat Application | Strengthening exercises  Balancing exercises  Muscle stimulation  TENS  Balancing exercises |  |  |

**Supplemental table 1: Common naturopathy treatments used by yoga and naturopathy physicians in India for managing peripheral polyneuropathies**
